# Extra Virgin Olive Oil Modulates Vasodilator Enzyme Level by Repairing Angiogenesis Function in Rat Model of Preeclampsia 

**Published:** 2020-03

**Authors:** Yulia Silvani, Agnestia Naning Dian Lovita, Afniari Maharani, I Wayan Arsana Wiyasa, Hidayat Sujuti, Retty Ratnawati, Tri Yudani Mardining Raras

**Affiliations:** 1 Midwifery Department, Faculty of Medicine, University of Brawijaya, Malang, Indonesia; 2 Department of Obstetrics and Gynecology, Dr. Saiful Anwar General Hospital, Malang, Indonesia; 3 Department of Biochemistry-Molecular Biology, Faculty of Medicine, University of Brawijaya, Malang, Indonesia; 4 Department of Physiology, Faculty of Medicine University of Brawijaya, Malang, Indonesia

**Keywords:** Preeclampsia, Extra Virgin Olive Oil, Vascular Endothelial Growth Factor, Endothelial Nitric Oxide Synthase, Angiogenic Factor

## Abstract

**Objective:** This study aimed to determine the effect of Extra Virgin Olive Oil (EVOO) on vasodilator enzyme by repairing angiogenic function in rat model of preeclampsia.

**Materials and methods:** This research consisted of five groups; negative control (normal pregnant rats) group, positive control (preeclampsia rat model) group, preeclampsia rat model groups given EVOO in 3 different doses (0.5 ml/day, 1 ml/day, and 2 ml/day, respectively). Blood pressure measurements were carried out on day 12, 15, and 19 of pregnancy. After the rats were sacrificed, the placentas were collected to determine endothelial Nitric Oxide Synthase (eNOS) level of maternal plasma to determine soluble Fms-like Tyrosine Kinase 1 (sFlt-1) and Vascular Endothelial Growth Factor (VEGF) level.

**Results:** There were significant higher sFlt-1 level (p < 0.001), lower VEGF level (p = 0.009), and lower eNOS level (p = 0.034) between negative and positive control groups. After EVOO administration, sFlt-1 level was lower in dose 1 and 2 groups but higher in dose 3 group in accordance with VEGF and eNOS levels that were increasing both in dose 1 and dose 2 groups but decreasing in dose 3. There were significant differences between positive control and dose 1 (p = 0.015) and dose 2 (p = 0.001) in sFlt-1 level. None of all dose groups were statistically different with positive control group in VEGF level (dose 1 p = 0.601; dose 2 p = 0.297; dose 3 p = 0.805). eNOS levels of all dose groups were statistically different from that of the positive control group (dose 1 p = 0.014; dose 2 p = 0.001; dose 3 p = 0.024).

**Conclusion:** Administration of EVOO modulates eNOS as vasodilator enzyme by repairing the angiogenic function indicated by decreased sFlt-1 level and increased VEGF in rat model of preeclampsia.

## Introduction

Preeclampsia is persistent hypertension accompanied by signs of high blood pressure and proteinuria in the second trimester of pregnancy (1). Several factors are thought to be the cause of preeclampsia, including abnormal genetic variations, trophoblast abnormalities, defense mechanisms of the immune system, and disorders of endothelial cells (2). Meanwhile, according to Sanjay and Girija, the pathophysiology of preeclampsia is affected by disorders of uteroplacental perfusion, oxidative stress, interference with nitric oxide pathways, changes in balance between angiogenic and antiangiogenic factors, lipid peroxidation, inflammatory factors due to poor placentation, fragments and placental microparticles due to many dead cells, autoantibodies, genetic factors, and immunological factors (3). In preeclampsia, there is a failure of the spiral artery remodeling process that is different from a normal pregnancy. Failure of the spiral artery remodeling in preeclampsia causes a decrease in uteroplacental perfusion and angiogenic and antiangiogenic imbalances that increase ischemia in the vascular placenta (4). Impairment of trophoblast invasion will disrupt the blood flow that results in ischemia and leads to hypoxia, oxidative stress, inflammation, and endothelial dysfunction (5). Placental ischemia is the cause of increased levels of soluble fms-like tyrosine kinase 1 (sFlt-1), soluble endoglin (sEng), and decreased vascular endothelial growth factor (VEGF) (6). sFlt-1 and sEng are antagonists of VEGF and PlGF (placental growth factor). sFlt-1 is antiangiogenic produced by the placenta under hypoxic conditions (4).

The process of endothelial dysfunction increases the elongation of sFlt-1 (sVEGFR-1), which is an endogenous inhibitor of VEGFR-1 and VEGF or PLGF bonds (7). Under normal circumstances, VEGFR-1 bonds with PLGF or VEGF to increase VEGFR-2 phosphorylation. The function of VEGFR-2 is activating PLC-gamma and PI3K. PLC-gamma will activate PKC through the formation of diacylglycerol and increase intracellular calcium concentration. CCP will produce eNOS. The adapter Shb molecule binds to Tyr1175 that activates PI3K, then AKT produces eNOS and gives rise to vascular permeability, cell migration, and cell survival (8). In the condition of preeclampsia, the above process is hampered due to the high sFlt-1 so that the process of eNOS formation is disrupted, Nitric Oxide (NO) decreases, and blood pressure increases.

EVOO acts as an antioxidant by inhibiting NADPH Oxidase so that it can reduce the production of free radicals, thereby repairing vascular endothelial damage. The vascular endothelial repair will reduce sFlt-1 level; thus, the bond between Flt1 and VEGF increases. In turn, the bond between Flt1 and VEGF will increase eNOS production through PLC-gamma and PI3K lines (8). Besides, the polyphenols contained in EVOO modulate the activation of the transcription factor of Nuclear Factor (Erythroid-Derived 2)-Like 2 (Nrf2) that plays a role in the regulation of angiogenic, resulting in an increase in VEGF level  (9). This study aimed to find out the role of EVOO to modulate vasodilator enzyme by repairing angiogenic function in the rat model of preeclampsia indicated by decreased sFlt-1 level, increased VEGF level, and increased eNOS level.

## Materials and methods

***Animal model*** ***: ***This research was in vivo laboratory research with Posttest-Only Control Group design. There were five groups in this study, with four rats in each group (10). The groups consist of negative control group that was normal pregnant rats; the positive control group that was preeclampsia pregnant rat model; and treatment groups 1, 2, and 3 that were preeclampsia rats given EVOO in three different doses (0,5 mL/day, 1 mL/day, and 2 mL/day, respectively) (11)*.*The next day after mating was determined as the first day of pregnancy. Rats were sacrificed on day 19 of pregnancy. The sample used in the study was placenta and plasma. The research was conducted in the Laboratory of Bioscience Brawijaya University, Laboratory of Physiology and Laboratory of Biomolecular Biochemistry, Faculty of Medicine Brawijaya University, Indonesia.

All procedures involving animals performed in studies was approved by the Ethics Committee Faculty of Medicine University of Brawijaya, Malang, Indonesia (ethical code: 73/EC/KEPK-S2/02/2019).

***Preeclampsia induction and EVOO administration***: The induction of preeclampsia was done using NOS inhibitors, L-NAME (C7H15N5O4 HCl) from Sigma-Aldrich (Merck KGaA, Darmstadt, Germany)    (12). Intraperitoneal injection of 125 mg/kg body weight L-NAME was given from day 13 to day 18 of pregnancy    (12) (13). Preeclampsia rat model was determined by the increased blood pressure above 140/90 mmHg. Meanwhile, EVOO was given per oral gavage using feeding tube from day 1 to day 18 of pregnancy.

***Blood pressure examination: ***Blood pressure examination was carried out by using non-invasive blood pressure measurement (CODA®, Kent Scientific Corporation) on day 12, day 15, and day 19 of pregnancy.

***sFlt-1, VEGF and eNOS level measurement: ***Rat plasma sample was used to measure thesFlt-1 level using rat ELISA kit (Elabscience catalog number E-EL-R0911) and the VEGF level using rat ELISA kit (MyBiosource catalog number MBS775506). Meanwhile, rat placental tissue sample was used to measure the eNOS level by using rat ELISA kit (Elabscience catalog number E-EL-R0367).

***Data analysis: ***The One-way ANOVA test was used to determine the mean difference among groups and continued by Multiple Comparison using Duncan and LSD tests to assess the differences among subgroups. Data were analyzed using SPSS 25 software.

## Results

**Figure 1:**
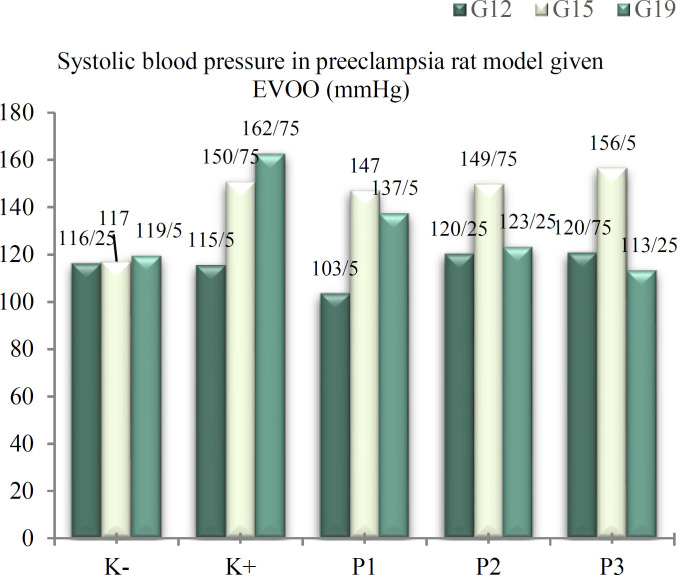
Systolic blood pressure in preeclampsia rat model given EVOO

***Blood pressure examination*** **: **Systolic blood pressure in all groups was under 140 mmHg before L-NAME injection. Systolic blood pressure increased in to > 140 mmHg after L-NAME injection (G15) in the positive control, dose 1, dose 2 and dose 3 groups (p < 0.001). On day 19 of pregnancy, systolic blood pressure decreased into < 140 mmHg after EVOO administration in all dose groups (p < 0.001) (Figure 1).

Systolic blood pressure on day 12 of pregnancy (G12), day 15 of pregnancy (G15), and day 19 of pregnancy (G19). Negative control group (K-), positive control group (K+), EVOO 0.5 mL/day (D1), EVOO 1 mL/day (D3), and EVOO 2 mL/day (D3).

**Figure 2:**
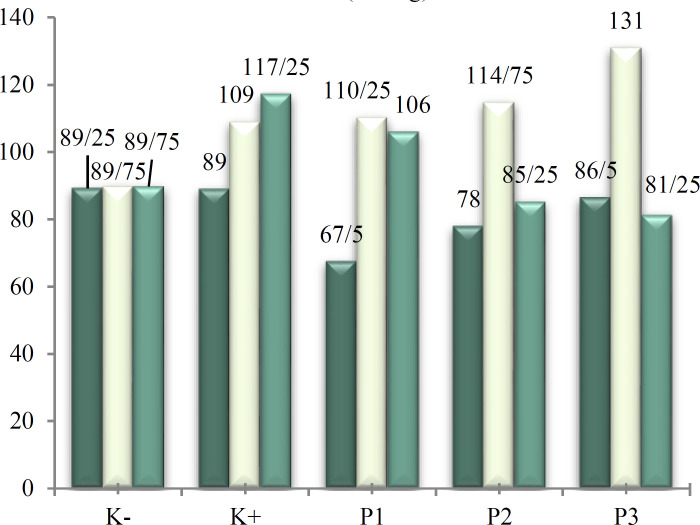
Diastolic blood pressure in preeclampsia rat model given EVOO

Diastolic blood pressures in all groups were under 90 mmHg before L-NAME injection. Diastolic blood pressure increased in to > 90 mmHg after L-NAME injection (G15) in positive control, dose 1, dose 2 and dose 3 groups (p < 0.001). On day 19 of pregnancy, diastolic blood pressure decreased after EVOO administration in all dose groups, although it did not reach < 90 mmHg in dose 1 (p = 0.001) (Figure 2).

Diastolic blood pressure on day 12 of pregnancy (G12), day 15 of pregnancy (G15), and day 19 of pregnancy (G19). Negative control group (K-), positive control group (K+), EVOO 0.5 mL/day (D1), EVOO 1 mL/day (D3), and EVOO 2 mL/day (D3).

***Effect of EVOO on*** ***sFlt-1 level in rat model of preeclampsia: ***Our data showed a significant higher sFlt-1 level in positive control group compared to that of the negative control group (p < 0.001). After EVOO administration, the sFlt-1 level decreased in dose 1 and dose 2 groups but increased in dose 3. Dose 1 (p = 0.015) and dose 2 (p = 0.001) groups were statistically different from the positive control group (Table 1).

**Table 1:** Mean of sFlt-1 level in pregnant rat model of preeclampsia

**Groups**	**sFlt-1 (mean ± SD)**	**P-value**
K (-)	0.223 ± 0.053	0.002*
K (+)	0.502 ± 0.054	
D1	0.338 ± 0.023	
D2	0.244 ± 0.033	
D3	0.408 ± 0.170	

sFlt-1 level (ng/dL) in pregnant rat model of preeclampsia was given EVOO on day 19 of pregnancy in negative control group (K -), positive control group (K +), EVOO 0.5 mL/day (D1), EVOO 1 mL/day (D2), and EVOO 2 mL/day (D3).

* One-way ANOVA test

***Effect of EVOO on*** ***VEGF level in rat model of preeclampsia: ***Our data showed a significant lower VEGF level in the positive control group compared to that in the negative control group (p = 0.009). After EVOO administration, the VEGF level increased in dose 1 and dose 2 groups but decreased in dose 3. None of the dose groups were statistically different from the positive control group (dose 1 p = 0.601; dose 2 p = 0.297; dose 3 p = 0.805) (Table 2).

**Table 2:** Mean of VEGF level in pregnant rat model of preeclampsia

**Groups**	**VEGF (mean ± SD)**	**P-value**
K (-)	279.00 ± 36.45	0.039*
K (+)	224.02 ± 27.56	
D1	233.86 ± 24.93	
D2	243.93 ± 18.46	
D3	219.38 ± 18.71	

VEGF level (pg/mL) in pregnant rat model of preeclampsia given EVOO onday19of pregnancy in negative control group (K -), positive control group (K +), EVOO 0.5 mL/day (D1), EVOO 1 mL/day (D2), and EVOO 2 mL/day (D3).

* One-way ANOVA test

***Effect of EVOO on*** ***eNOS level in rat model of preeclampsia: ***Our data showed a significant lower eNOS level in the positive control group compared to that in the negative control group (p = 0.034). After EVOO administration, the eNOS level increased in dose 1 and dose 2 groups but decreased in dose 3. All of doses groups were statistically different from the positive control group (dose 1 p = 0.014; dose 2 p = 0.001; dose 3 p = 0.024) (Table 3).

**Table 3:** Mean of eNOS level in pregnant rat model of preeclampsia

**Groups**	**eNOS (mean ± SD)**	**P value**
K (-)	158.54 ± 84.98	0.011*
K (+)	74.41 ± 35.61	
D1	175.39 ± 19.44	
D2	229.72 ± 60.69	
D3	165.04 ± 21.75	

eNOS level (pg/mL) in pregnant rat model of preeclampsia given EVOO on day 19of pregnancy in negative control group (K -), positive control group (K +), EVOO 0.5 mL/day (D1), EVOO 1 mL/day (D2), and EVOO 2 mL/day (D3).

* One-way ANOVA test

## Discussion

In this study, the effects of EVOO administration on sFlt-1 level, VEGF level, and eNOS level in rat model of preeclampsia were evaluated. The results showed that in preeclampsia rats, there were increased sFlt-1 level, decreased VEGF level, and decreased eNOS level. These results were in line with previous studies (14-17). The preparation of preeclampsia experimental animals made by Zhu et al by using of L-NAME in rats aged 8 weeks and started from day 13 of pregnancy was reported successful marked by the emergence of hypertension, proteinuria, high fetal death rates, and the presence of growth disorders in the fetus (14). Zhu et al also confirm that changes in NO synthesis are one of the triggering factors of preeclampsia (14).

The administration of EVOO has positive effects on health, and this is associated with the composition of special fatty acids, high content of oleic acid, polyunsaturated essential fatty acids (PUFA), low ratio of PUFA n-6 / PUFA n-3, and high bioactive compounds including phenols, sterols, hydrocarbons (squalene), vitamins (α- and γ-tocopherol), β-carotene, and other phytosterols (18, 19). Research conducted by Cárdeno A. et al. and Sánchez-Fidalgo S et al. report that phenolic compounds have broad-spectrum bioactive properties, including antioxidants, free-radicals scavenging, anti-inflammatory effects and chemo preventive effects (19).

The normal placentation process depends on the balance of expression of angiogenic and antiangiogenic factors during pregnancy. VEGF as an angiogenic factor supports the normal placentation process by binding to cellular receptors, including Flt-1 (VEGFR1), Flk-1 (VEGFR2), and Flt-4 (VEGFR3). In preeclampsia, there is an increase of sFlt-1 level in the placenta as well as in the maternal serum. Under normal circumstances, VEGFR-1 or Flt-1 bond on PLGF and VEGF will increase VGFR-2phosphorylation. The function of VEGFR-2 is to activate PLC-gamma and PI3K. PLC-gamma will activate PKC through the formation of diacylglycerol and increase intracellular calcium concentration. CCP will produce eNOS. Shb molecule adapter binds to Tyr1175,which activates PI3K,and then AKT produces eNOS and gives rise to vascular permeability, cell migration, and cell survival (8). In preeclampsia condition, the process above is hampered due to high Sflt-1 and low VEGF, so the process of forming eNOS is interrupted.

EVOO can reduce sFlt-1 level, increase VEGF level, and eNOS level in preeclampsia rats. This is because EVOO polyphenols modulate the activation of the transcription of Nuclear Factor (Erythroid-Derived 2)-Like 2 (Nrf2), which plays a role in angiogenic regulation. Nrf2 is a transcription factor of the gene that encodes Heme oxygenase-1 (HO-1). HO-1 functions to degrade heme into free iron, CO, and biliverdin. One of the results of HO-1 degradation, namely CO, initiate another transcription factor, Hypoxia-inducible Factor-1α (HIF-1α). HIF-1α binds to the promoter side in the VEGF coding genes which then go through a transcription process to form VEGF mRNA. VEGF mRNA is translated into various amino acid products making up VEGF. It is through Nrf2 / HO-1 activation pathway where EVOO polyphenols increase the VEGF levels (9, 20, 21).

The antioxidants in EVOO can delay the oxidation process. In this case, the main antioxidant that inhibits the oxidation process in EVOO is OP (Olive Phenols), which breaks down the chain by donating hydrogen radicals to alkyl peroxyl radicals, which are produced by lipid oxidation and stable derivative formation during the reaction. Research on the antioxidant activity of EVOO polyphenols conducted by Carrasco-Pancorbo et al confirms the possibility that phenol acts as a hydrogen donor, and oxidation in EVOO is inhibited by an increase in the number of hydroxyl groups in the OPs’ structure (22). It was also reported that compounds related to the function of o-dihydroxyl have high antioxidant activity because the formation of intramolecular hydrogen bonds was observed during the reaction with free radicals. In addition, phenol O-H bonds are weakened by substituents which donate electrons to the "ortho" position and stabilize phenoxyl radicals.

In preeclampsia rats given EVOO dose 3, there were increasing sFlt-1 level, decreasing VEGF level, and decreasing eNOS level. It is possible because the anti-inflammatory properties in olive oil can change into pro-inflammatory, which may cause harm to health. Experimental studies have shown that EVOO hydroxyl tyrosol gives a pro-inflammatory effect that increases the area of atherosclerotic lesions, and the expression of Mac-1 circular monocytes triggers monocyte recruitment in inflammatory processes (23). Increased monocyte recruitment in the inflammatory process converts monocytes into activated macrophages which increase the synthesis of TNF-α (24). TNF-α stimulates the expression of sFlt-1 and binds to VEGF, which causes a decrease in free VEGF levels (25-27). Dose 3 (2 ml/day) in this study seems to have a pro-inflammatory effect in pregnant preeclampsia rats that results in decreases in VEGF and eNOS and an increase in sFlt-1.

The administration of a higher dose of Extra Virgin Olive Oil (EVOO) also allows for changes in the content of some antioxidants that provide a pro-oxidant effect. In a study conducted by Maurya and Devasagayam, oleuropein and hydroxyl tyrosol contained in EVOO are reported to have a pro-oxidative effect due to inhibitory activity of iron and copper (28). This reduced metal catalyzes the production of OH radicals via the Fenton reaction. Another study related to antioxidant and pro-oxidant activity concludes that the ability of polyphenols to act as antioxidants or pro-oxidant in in-vitro and in-vivo systems depends on several factors such as concentration and structure (29).

In this research, phytochemical test was not conducted to determine the types and active compounds (polyphenols) on brand "B" EVOO which has the most dominant antioxidant. For further research, we suggest conducting toxicity tests of EVOO doses to determine the effects on mother and fetus, so the results can be used as basis for EVOO administration to pregnant women.

## Conclusion

Administration of EVOO modulates eNOS as vasodilator enzyme by repairing angiogenic function indicated by decreased sFlt-1 level and increased VEGF in rat model of preeclampsia.
